# Patterns of multimorbidity across obesity severity and fat distribution in Anhui, China: a community-based study

**DOI:** 10.3389/fendo.2025.1652678

**Published:** 2025-09-10

**Authors:** Keyi Gu, Weiqiang Wang, Weizhuo Yi, Handong Gu, Xiaoya Fu, Fei Yang

**Affiliations:** ^1^ Department of General Medicine, Suzhou Hospital affiliated to Anhui Medical University (Suzhou Municipal Hospital of Anhui), Suzhou, China; ^2^ Anhui Medical University, Hefei, China; ^3^ Department of Epidemiology and Health Statistics, School of Public Health, Anhui Medical University, Hefei, China

**Keywords:** multimorbidity, body mass index, fat distribution, central obesity, association rule mining

## Abstract

**Introduction:**

Obesity and multimorbidity are prevalent worldwide. However, the relationships of obesity severity and fat distribution with multimorbidity patterns among the Chinese population are still unclear. We sought to investigate multimorbidity patterns among people with various obesity severity and fat distribution in Anhui, China.

**Methods:**

We used cross-sectional data including 123,148 adults aged 35–76 years in 12 districts from Anhui Province, China. Multimorbidity referred to the presence of at least two chronic conditions from a defined set of nine. We used logistic regression models, stratified by gender, to analyze the associations of different obesity severity and fat distribution with the risk of multimorbidity by adjusting for confounders of age, region, marriage, education level, annual income, insurance, smoking, drinking, rational diet, weight control, physical exercise, adequate sleep and regular checkup. Subgroup and interaction analyses examined how varying obesity severity and fat distribution relate to multimorbidity risk. Association rule mining (ARM) utilized the Apriori algorithm to analyze disease combinations under different obesity subgroups in males and females.

**Results:**

Multimorbidity occurred in 10.3%(n=12,644) of the participants, with 10.7%(n=5,324) in males and 9.96% (n=7,320) in females, and the majority (80.5%, n=10,177) had two chronic diseases. Compared to normal-weight participants, there were progressively higher odds of multimorbidity in overweight, mild, moderate, and severe obesity in both males and females (P for trend <0.001). Individuals with general obesity (male: OR = 1.366, 95% CI: 1.234–1.513; female: OR = 1.315, 95% CI: 1.197–1.445), central obesity (male: OR = 2.168, 95% CI: 1.857–2.532; female: OR = 1.567, 95% CI: 1.401–1.752), or compound obesity (male: OR = 2.223, 95% CI: 1.996–2.476; female: OR = 1.998, 95% CI: 1.822–2.190) had significantly higher multimorbidity rates than their non-obese counterparts. Subgroup analysis and interaction analysis results showed that males, people aged < 60 years, and smokers may worsen the effects of obesity on multimorbidity. ARM revealed that the disease cluster comprising diabetes, hypertension, and dyslipidemia exhibited the strongest association. Notably, males with severe obesity face an elevated risk of cardiovascular metabolic comorbidity.

**Conclusions:**

Both overweight and obesity are independent risk factors for multimorbidity, and males exhibit significantly higher multimorbidity risks than females. Individuals with obesity are more vulnerable to multiple coexisting conditions such as diabetes, hypertension, and dyslipidemia. Therefore, adopting health management and intervention measures for obesity individuals can help control multimorbidity.

## 1 Introduction

Obesity remains a significant worldwide health issue, with China experiencing rising rates of obesity and related chronic diseases. Epidemiological data highlight a dramatic rise in obesity prevalence among Chinese adults, from 8.1% in 2018 to 10.8% in 2022 (1), and the combined overweight and obesity rate is estimated to climb to 65.3% by 2030 (2). Obesity independently elevates the likelihood of numerous chronic illnesses such as high blood pressure, dyslipidemia, diabetes, heart-related conditions, and even cancer (3–5). Additionally, it is strongly associated with multimorbidity (6). Multimorbidity refers to the concurrent existence of at least two chronic conditions in a single patient and poses an escalating clinical and public health challenge. When contrasted with single chronic disease patients, multimorbidity individuals experience increased risks of premature mortality, lower quality of life, higher healthcare costs, and polypharmacy (7). Therefore, early management and intervention for individuals with obesity are crucial for mitigating the onset and progression of multimorbidity.

Obesity screening typically relies on body mass index (BMI) as the conventional anthropometric assessment tool. However, body fat distribution can vary significantly even at the same BMI level. Visceral fat accumulation—characteristic of central obesity—has stronger pro-inflammatory and metabolic effects than subcutaneous fat and is closely linked to obesity-related multimorbidity (8). Previous studies have demonstrated that anthropometric parameters including BMI, waist circumference (WC), and waist-height proportion demonstrate that obesity substantially elevates the probability of developing multimorbidity (9, 10). However, the relationships of different fat distribution—namely, general obesity, central obesity, and compound obesity—with multimorbidity patterns remain unclear among Chinese adults.

Moreover, as a key province in central China, Anhui may exhibit distinct patterns of obesity prevalence, chronic disease, and multimorbidity due to its unique regional socioeconomic and dietary characteristics. However, research focusing on these regional elements is scarce. Therefore, this community-based study examined data from 123,148 participants across 12 regions in Anhui Province to fill this void, focusing on a general population not selected for specific health conditions. We aimed to investigate multimorbidity patterns among people with various obesity severity and fat distribution stratified by gender and to analyze specific combinations of chronic diseases within different obesity subgroups.

## 2 Materials and methods

### 2.1 Experimental design and subjects

This study utilized data from Anhui Province’s Cardiovascular Disease High-Risk Population Early Screening and Comprehensive Intervention Initiative. Between 2017 and 2021, the community-based program enrolled all eligible residents aged 35–76 years (n=133,178) from initial community health screening across 12 designated project sites. These sites included Wangjiang County (Anqing City), Huaiyuan County (Bengbu City), Dongzhi County (Chizhou City), Taihe County (Fuyang City), Feixi County (Hefei City), Yu’an District (Lu’an City), Huashan District (Ma’anshan City), Yongqiao District (Suzhou City), Wuhu County (Wuhu City), Ningguo City (Xuancheng City), Mengcheng County (Bozhou City), and Tongguan District (Tongling City). The project sites were selected to ensure reasonable regional representation and to include relatively complete resident records.

The inclusion criteria required participants to have resided at the project site for a minimum of six months during the preceding year to ensure they were permanent residents and to have signed an informed consent form. Participants with missing data on height, weight, or biochemical indicators were excluded from the analysis. Consequently, the investigation successfully recruited 123,148 eligible subjects over the course of the research.

### 2.2 Data collection

Custom-designed forms were administered through in-person interviews to collect information on factors such as age, gender, marital status, education level, residence, annual income, insurance status, and lifestyle. Study data were collected through face-to-face interviews using an electronic data collection system that recorded structured information on age, gender, marital status, education level, residence, annual income, insurance status, and lifestyle factors. Clinical examinations focused on essential anthropometric parameters including weight, height, and WC. Using a digital sphygmomanometer, systolic and diastolic pressures (SBP/DBP) along with heart rate (HR) were recorded, with multiple readings (minimum two per minute) averaged for accuracy. Blood samples were collected after fasting for 6–8 hours at the local community health service center. Fasting blood glucose (FPG) levels were measured using a BeneCheck (BK6-20MD) glucometer, while the lipid panel - including total cholesterol (TC), triglycerides (TG), high-density lipoprotein cholesterol (HDL-C), and low-density lipoprotein cholesterol (LDL-C) - was analyzed using a CardioChek P·A analyzer. All measurements were performed by trained physicians under standardized clinical operating conditions.

### 2.3 Assessments of covariates and multimorbidity

This research examined nine chronic diseases: hypertension, diabetes, dyslipidemia, angina, stroke, chronic obstructive pulmonary disease (COPD), renal or ureteral stones, myocardial infarction, and cancer—all identified through participants’ self-reported physician diagnoses. Participants were categorized as having multimorbidity when their medical history indicated diagnoses for two or more of the specified conditions.

Education levels were divided into two groups: secondary education or below versus higher secondary education and beyond. Tobacco and alcohol use was classified as “yes” or “no” based on current consumption status. Rational diet was defined as having three regular meals per day to ensure a balanced intake of various nutrients. Weight control referred to the regular monitoring of body weight along with conscious efforts to maintain or improve it. Physical exercise was characterized by exercising at least 3–5 times weekly. Adequate sleep was characterized by a consistent routine, including regular sleeping and eating schedules. Regular checkups were defined as annual health examinations or scheduled follow-ups for chronic conditions.

The study areas were classified into urban and rural regions based on China’s administrative hierarchy: urban areas referred to administratively demarcated city districts at the municipal and prefectural levels, while rural areas included towns and townships under county-level administration. Northern Anhui covers the territory located north of the Huaihe River, encompassing six prefecture-level cities: Suzhou, Huaibei, Bengbu, Fuyang, Huainan, and Bozhou. Central Anhui lies between the Huaihe and Yangtze Rivers, featuring cities such as Hefei, Lu’an, Chuzhou, and Anqing. Meanwhile, Anhui’s southern region extends across the Yangtze River’s southern bank, encompassing urban centers such as Huangshan, Wuhu, Ma’anshan, Tongling, Xuancheng, and Chizhou.

### 2.4 Assessment of obesity severity and fat distribution

Body mass index (BMI) was derived by dividing an individual’s body weight in kilograms by their height in meters squared. Subjects were categorized into four distinct groups according to their body fat distribution. Participants were classified as non-obesity group if their BMI was less than 28.0 kg/m², combined with WC measurements below 90.0 cm for men or 85.0 cm for women. A BMI of 28.0 kg/m² or higher was classified as general obesity (11). Central obesity was defined as a WC of 90.0 cm or more for men and 85.0 cm or more for women (12). The compound obesity group included those who exceeded both the BMI and WC criteria simultaneously for their respective ses.

The diagnostic criteria employed were derived from the latest Chinese guidelines for the diagnosis and treatment of obesity (13). Classification of BMI ranges as: <18.5 kg/m² for underweight, 18.5-23.9 kg/m² for normal, 24.0-27.9 kg/m² for overweight, and ≥28.0 kg/m² for obesity. Obesity severity was further classified as: mild obesity (BMI 28.0-< 32.5 kg/m²), moderate obesity (BMI 32.5–< 37.5 kg/m²), and severe obesity (BMI ≥ 37.5 kg/m²).

### 2.5 Statistical analysis

Statistical analyses were performed with R software (version 4.4.2) for all datasets. Statistical significance was defined as a two-tailed P-value < 0.05. Qualitative data were reported as adjusted proportions (%), while quantitative measurements were shown as mean ± SD. Chi-square tests were employed to evaluate qualitative measures, while regression models analyzed quantitative parameters to determine statistical significance. Logistic regression models examined how different obesity severity and fat distribution influenced multimorbidity likelihood, adjusting for potential confounding variables such as age, region, marriage, education level, annual income, insurance, smoking, drinking, rational diet, weight control, physical exercise, adequate sleep and regular checkup. Association rule mining (ARM) utilized the Apriori algorithm to analyze disease combinations under different obesity subgroups with three evaluation metrics: support (the frequency of a particular combination), confidence (the conditional probability of the consequent given the antecedent), and lift (the ratio of observed to expected support). We defined the minimum thresholds for ARM parameters as follows: support > 0.02 and confidence > 0.02. Lift was considered the primary indicator of rule significance.

## 3 Results

### 3.1 Demographic and clinical characteristics at baseline by gender

Participants had an average age of 57.7 years (SD = 10.0), with women comprising 59.7% (n = 73,481) of the cohort and rural residents accounting for 63.4% (n = 78,132), as detailed in **Supplementary Table 1**. The number (%) of participants with 0, 1, and ≥2 chronic diseases was 29,053 (58.5%), 15,290 (30.8%), and 5,324 (10.7%) in males (**Table 1**), and 45,848 (62.4%), 20,313 (27.6%), and 7,320 (10.0%) in females (**Table 2**), respectively. The results of the chi-square test indicated that age, region, marriage, education level, annual income, and lifestyle significantly influenced the number of chronic diseases in both genders (*P* < 0.001). Additionally, participants with multimorbidity had significantly higher BMI, WC, SBP, DBP, HR, TC, TG, and FPG, and substantially lower HDL-c than other groups in both males and females (*P* < 0.001). Notably, males exhibited substantially higher current rates of smoking (45.5%) and drinking (32.1%) compared to females (1.6% and 3.5%, respectively).

**Table 1:** Baseline characteristics of male participants by the number of chronic diseases.

Parameters	Overall	N of chronic diseases			*P* value
		0	1	≥2	
Number of participants (%)	49667	29053 (58.5%)	15290 (30.8%)	5324 (10.7%)	
Socio-demographic factors					
Age (years)	58.66 ± 10.15	56.97 ± 10.41	60.77 ± 9.37	61.80 ± 8.83	<0.001
Age groups (%)					<0.001
(35-45]	11.8	15.8	6.9	4.5	
(45-60]	39.6	42.4	35.9	34.7	
≥60	48.6	41.8	57.2	60.8	
Region (%)					<0.001
Northern Anhui	24.1	25.1	22.0	24.5	
Central Anhui	32.1	34.5	29.9	25.1	
Southern Anhui	43.9	40.5	48.1	50.4	
Rural (%)	61.5	60.2	64.2	60.3	<0.001
Married (%)	92.3	92.9	91.4	92.0	<0.001
High school or above (%)	14.3	13.8	13.8	18.0	<0.001
Annual income≥50k (%)	21.8	22.9	19.9	21.6	<0.001
Have insurance (%)	99.5	99.4	99.6	99.6	0.025
Lifestyle factors					
Smoking (%)	45.5	45.4	47.3	41.0	<0.001
Drinking (%)	32.1	29.4	37.2	32.6	<0.001
Rational diet (%)	27.6	28.1	26.1	29.1	<0.001
Weight control (%)	44.5	37.7	51.5	61.4	<0.001
Physical exercise (%)	58.6	53.1	64.4	72.0	<0.001
Adequate sleep (%)	24.1	24.2	23.6	25.1	0.082
Regular checkup (%)	20.6	19.7	20.8	24.7	<0.001
Clinical indicators					
BMI	24.47 ± 3.21	23.98 ± 3.05	24.98 ± 3.28	25.67 ± 3.28	<0.001
WC (cm)	85.83 ± 9.25	84.32 ± 8.83	87.35 ± 9.41	89.71 ± 9.23	<0.001
SBP (mmHg)	137.52 ± 18.62	132.73 ± 16.44	143.65 ± 19.55	146.04 ± 18.85	<0.001
DBP (mmHg)	82.54 ± 10.74	80.41 ± 9.80	85.39 ± 11.32	85.95 ± 11.17	<0.001
HR (bpm)	72.65 ± 10.43	72.18 ± 10.00	73.05 ± 10.80	74.07 ± 11.42	<0.001
TC (mmol/L)	4.37 ± 0.95	4.34 ± 0.92	4.43 ± 0.97	4.41 ± 1.06	<0.001
LDL-c (mmol/L)	2.31 ± 0.83	2.29 ± 0.80	2.34 ± 0.84	2.31 ± 0.92	<0.001
HDL-c (mmol/L)	1.41 ± 0.40	1.44 ± 0.40	1.40 ± 0.39	1.33 ± 0.37	<0.001
TG (mmol/L)	1.51 ± 0.84	1.45 ± 0.80	1.56 ± 0.85	1.69 ± 0.92	<0.001
FPG (mmol/L)	6.13 ± 1.73	5.79 ± 1.25	6.36 ± 1.93	7.32 ± 2.58	<0.001

Mean ± SD for continuous variables: the p-value was calculated by the weighted linear regression model. (%) for categorical variables: the p-value was calculated by the weighted chi-square test. BMI, body mass index; WC, waist circumference; SBP, systolic blood pressure; DBP, diastolic blood pressure; HR, heart rate; TC, total cholesterol; LDL-c, low-density lipoprotein cholesterol; HDL-c, high-density lipoprotein cholesterol; TG, triglycerides; FPG, fasting blood glucose.

**Table 2:** Baseline characteristics of female participants by the number of chronic diseases.

Parameters	Overall	N of chronic diseases			*P* value
		0	1	≥2	
Number of participants (%)	73481	45848(62.4%)	20313 (27.6%)	7320 (10.0%)	
Socio-demographic factors					
Age (years)	57.07 ± 9.87	54.90 ± 9.87	60.09 ± 8.91	62.25 ± 8.12	<0.001
Age groups (%)					<0.001
(35-45]	12.9	17.9	5.3	2.5	
(45-60]	46.8	50.6	42.6	34.7	
≥60	40.3	31.5	52.1	62.8	
Region (%)					<0.001
Northern Anhui	27.7	27.9	26.3	30.2	
Central Anhui	31.2	33.1	29.7	23.3	
Southern Anhui	41.1	39.0	44.0	46.5	
Rural (%)	64.8	62.8	68.3	67.2	<0.001
Married (%)	89.1	90.8	86.5	85.0	<0.001
High school or above (%)	7.8	9.2	5.4	5.8	<0.001
Annual income≥50k (%)	18.8	21.0	15.6	14.3	<0.001
Have insurance (%)	99.5	99.4	99.5	99.4	0.806
Lifestyle factors					
Smoking (%)	1.6	1.5	1.7	1.9	0.022
Drinking (%)	3.5	3.3	3.8	3.3	0.001
Rational diet (%)	31.0	32.0	29.2	29.6	<0.001
Weight control (%)	46.0	39.6	54.7	62.0	<0.001
Physical exercise (%)	62.5	57.7	69.0	74.1	<0.001
Adequate sleep (%)	26.8	27.7	25.6	24.7	<0.001
Regular checkup (%)	21.7	21.2	21.5	24.6	<0.001
Clinical indicators					
BMI	24.64 ± 3.48	24.11 ± 3.29	25.35 ± 3.59	25.97 ± 3.61	<0.001
WC (cm)	82.98 ± 9.32	81.39 ± 8.82	85.06 ± 9.50	87.11 ± 9.51	<0.001
SBP (mmHg)	136.55 ± 20.32	130.91 ± 17.75	145.03 ± 20.94	148.31 ± 20.46	<0.001
DBP (mmHg)	79.10 ± 10.46	77.21 ± 9.68	82.30 ± 11.00	82.07 ± 10.78	<0.001
HR (bpm)	74.46 ± 9.79	74.17 ± 9.39	74.79 ± 10.34	75.36 ± 10.54	<0.001
TC (mmol/L)	4.75 ± 1.03	4.65 ± 0.99	4.89 ± 1.04	4.92 ± 1.12	<0.001
LDL-c (mmol/L)	2.52 ± 0.88	2.46 ± 0.85	2.62 ± 0.90	2.61 ± 0.97	<0.001
HDL-c (mmol/L)	1.53 ± 0.39	1.55 ± 0.39	1.52 ± 0.38	1.48 ± 0.37	<0.001
TG (mmol/L)	1.60 ± 0.80	1.52 ± 0.77	1.69 ± 0.82	1.85 ± 0.88	<0.001
FPG (mmol/L)	6.10 ± 1.74	5.74 ± 1.19	6.39 ± 1.98	7.50 ± 2.75	<0.001

Mean ± SD for continuous variables: the p-value was calculated by the weighted linear regression model. (%) for categorical variables: the p-value was calculated by the weighted chi-square test. BMI, body mass index; WC, waist circumference; SBP, systolic blood pressure; DBP, diastolic blood pressure; HR, heart rate; TC, total cholesterol; LDL-c, low-density lipoprotein cholesterol; HDL-c, high-density lipoprotein cholesterol; TG, triglycerides; FPG, fasting blood glucose.

**Figure 1:**
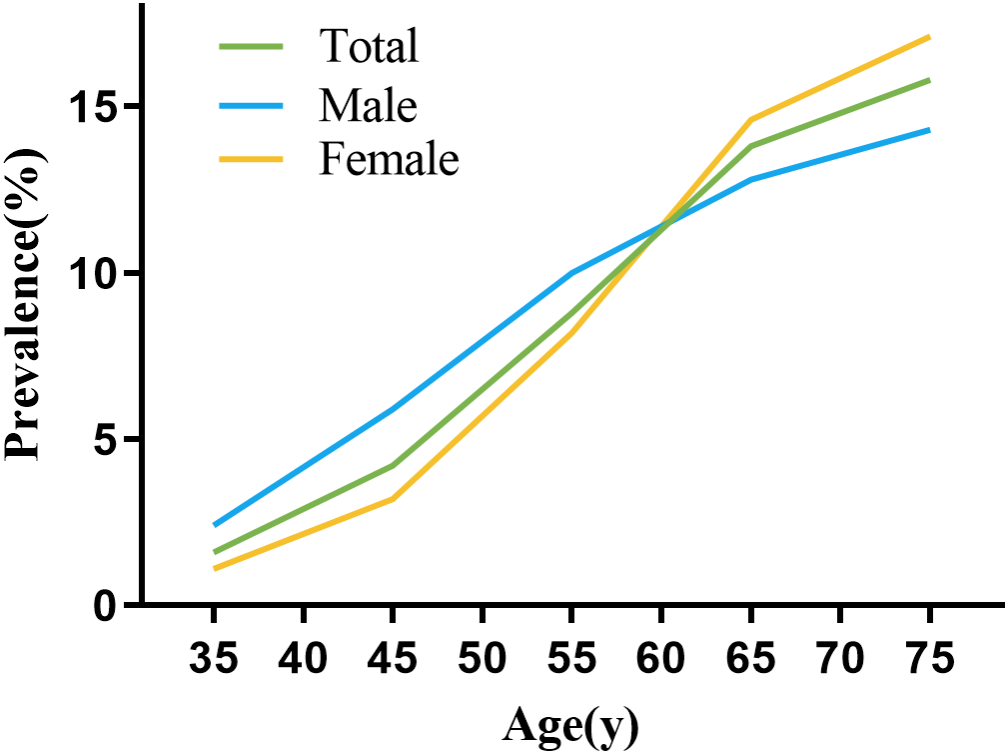
Prevalence of multimorbidity in different ages and genders.

**Figure 1** illustrates the frequency of multimorbidity among various age categories and by gender, revealing a progressive increase with advancing age. Notably, a gender reversal in prevalence occurs around the age of 60: males exhibit higher rates of multimorbidity before this age, while females show higher rates thereafter.

**Figure 2:**
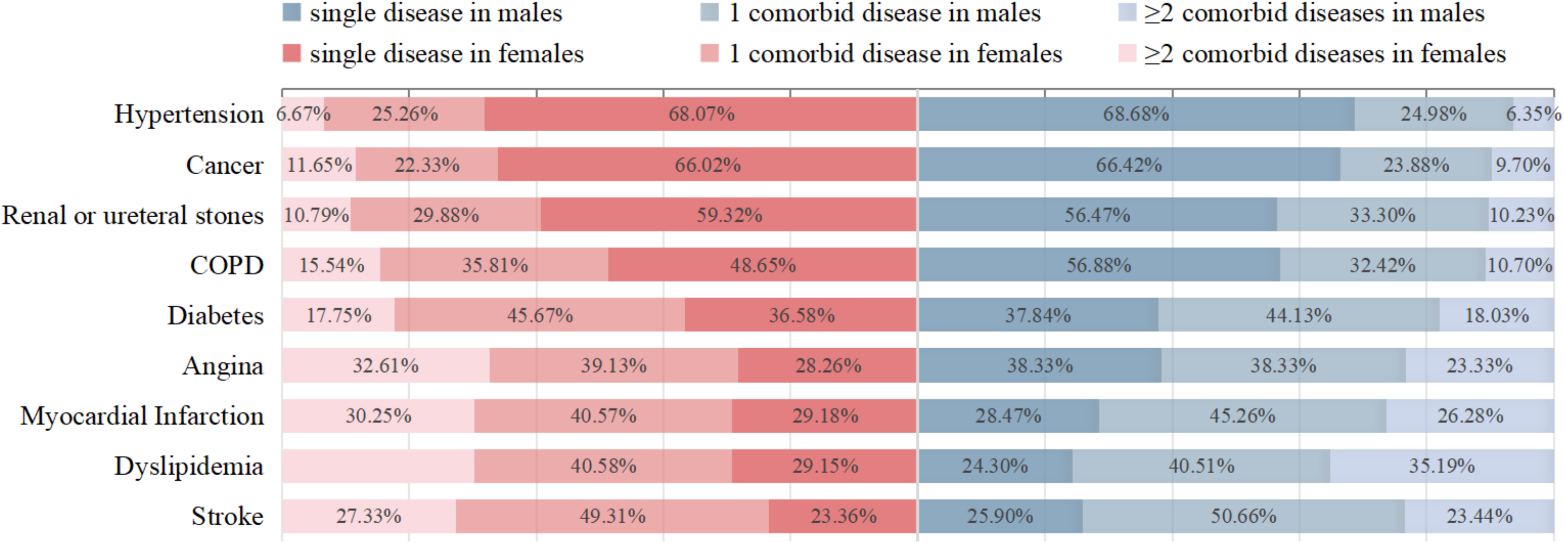
The prevalence percentage of multimorbidity of nine chronic diseases by gender.

The prevalence of multimorbidity is presented in **Table 3**; **Figure 2**. Among the nine chronic diseases analyzed, hypertension showed the highest individual prevalence at 68.68% (10,701/15,582) in males and 68.07% (14,295/21,000) in females, followed by cancer, renal or ureteral stones, and COPD (**Figure 2**). The most common type of multimorbidity involving two diseases was stroke (50.66% [927/1,830] in males; 49.31% [1,043/2,115] in females) and the most common type involving three diseases was dyslipidemia (35.19% [569/1,617] in males; 30.27% [924/3,053] in females). Among males, dyslipidemia was most frequently associated with multimorbidity (75.70%) and exhibited the highest mean multimorbidity count. Among females, stroke was most likely accompanied by multimorbidity (76.64%), while angina pectoris exhibited the highest average number of multimorbidity (**Table 3**).

**Table 3:** Prevalence of multimorbidity and average number of multimorbidity for nine chronic diseases by gender.

Chronic disease	Male			Female		
	N	Prevalence (%)^a^	Mean ± SD^b^	N	Prevalence (%)^a^	Mean ± SD^b^
Hypertension	15582	31.32	2.23	21000	31.93	2.24
Diabetes	4043	62.16	2.33	6350	63.42	2.33
Renal or ureteral stones	3216	43.53	2.28	3363	40.68	2.32
Dyslipidemia	1617	75.70	2.55	3053	70.85	2.51
Stroke	1830	74.10	2.38	2115	76.64	2.45
Myocardial Infarction	274	71.53	2.52	281	70.82	2.61
COPD	327	43.12	2.30	148	51.35	2.36
Cancer	134	33.58	2.33	206	33.98	2.44
Angina	60	61.67	2.54	92	71.74	2.67

a Weighted prevalence of multimorbidity among those with each chronic disease. ^b^Weighted mean and unweighted standard deviation of number of multimorbidity among those with each chronic disease.

### 3.2 Association of different obesity severity and fat distribution with the risk of multimorbidity

**Table 4** summarizes the relationships between different obesity severity and risk of multimorbidity by gender. Univariate logistic regression analysis (model I) revealed that relative to normal-weight participants, those classified as overweight or with mild, moderate, or severe obesity exhibited progressively higher risks of developing multimorbidity in both males and females (*P* for trend <0.001). Following covariate adjustment including age, region, marriage, high school or above, annual income, insurance, smoking, drinking, rational diet, weight control, physical exercise, adequate sleep, and regular checkups (model II), the adjusted odds ratios (ORs) in males are overweight (OR=1.454 95% CI: 1.320-1.600), mild obesity (OR=2.083 95% CI: 1.841-2.357), moderate obesity (OR=2.989 95% CI: 2.377-3.760), and severe obesity (OR=3.755 95% CI: 1.913-7.369), with a slightly higher risk of multimorbidity compared to females across different obesity severity.

**Table 4:** Multivariable OR (95% CI) for the risk of multimorbidity according to obesity severity by gender.

Characteristic	Male		Female	
	OR (95% CI)	*P* value	OR (95% CI)	*P* value
Model I				
Normal	–	–	–	–
Underweight	0.679 (0.515,0.895)	0.006	0.710 (0.566,0.889)	0.003
Overweight	1.837 (1.718,1.963)	<0.001	1.732 (1.636,1.833)	<0.001
Mild obesity	2.683 (2.466,2.919)	<0.001	2.710 (2.530,2.903)	<0.001
Moderate obesity	3.497 (2.847,4.296)	<0.001	3.456 (3.024,3.948)	<0.001
Severe obesity	4.047 (2.111,7.758)	<0.001	3.034 (2.066,4.456)	<0.001
*P* for trend	<0.001		<0.001	
Model II				
Normal	–	–	–	–
Underweight	0.601 (0.455,0.794)	<0.001	0.583 (0.464,0.734)	<0.001
Overweight	1.454 (1.320,1.600)	<0.001	1.422 (1.310,1.544)	<0.001
Mild obesity	2.083 (1.841,2.357)	<0.001	2.065 (1.857,2.295)	<0.001
Moderate obesity	2.989 (2.377,3.760)	<0.001	2.751 (2.352,3.218)	<0.001
Severe obesity	3.755 (1.913,7.369)	<0.001	2.643 (1.762,3.966)	<0.001
*P* for trend	<0.001		<0.001	

Model I, unadjusted; model II, adjusted for age, female, region, marriage, high school or above, annual income, insurance, smoking, drinking, rational diet, weight control, physical exercise, adequate sleep and regular checkup.

**Table 5** summarizes the relationships between fat distribution and risk of multimorbidity, stratified by gender. Compared to the non-obesity reference group, all obesity subtypes significantly increased the risk for multimorbidity (all *P* < 0.001), with compound obesity showing the highest ORs in both males (OR = 2.773, 95% CI: 2.589–2.970 in Model I) and females (OR = 2.729, 95% CI: 2.573–2.894 in Model I). Following covariate adjustment in Model II, multivariate analysis demonstrated that general obesity, central obesity, and compound obesity remained significant risk factors for multimorbidity. Notably, central obesity exhibited a stronger effect in males (OR = 2.168, 95% CI: 1.857-2.532 in Model II) than in females (OR = 1.567, 95% CI: 1.401-1.752 in Model II).

**Table 5:** Multivariable OR (95% CI) for the risk of multimorbidity according to fat distribution by gender.

Characteristic	Male		Female	
	OR (95% CI)	*P* value	OR (95% CI)	*P* value
Model I				
Non-obesity	–	–	–	–
General obesity	1.590 (1.468,1.724)	<0.001	1.448 (1.341,1.563)	<0.001
Central obesity	2.166 (1.859,2.524)	<0.001	1.879 (1.684,2.098)	<0.001
Compound obesity	2.773 (2.589,2.970)	<0.001	2.729 (2.573,2.894)	<0.001
Model II				
Non-obesity	–	–	–	–
General obesity	1.366 (1.234,1.513)	<0.001	1.315 (1.197,1.445)	<0.001
Central obesity	2.168 (1.857,2.532)	<0.001	1.567 (1.401,1.752)	<0.001
Compound obesity	2.223 (1.996,2.476)	<0.001	1.998 (1.822,2.190)	<0.001

Model I, unadjusted; model II, adjusted for age, female, region, marriage, high school or above, annual income, insurance, smoking, drinking, rational diet, weight control, physical exercise, adequate sleep and regular checkup.

### 3.3 Subgroup analyses of the association of obesity severity and fat distribution with multimorbidity risk

**Figure 3:**
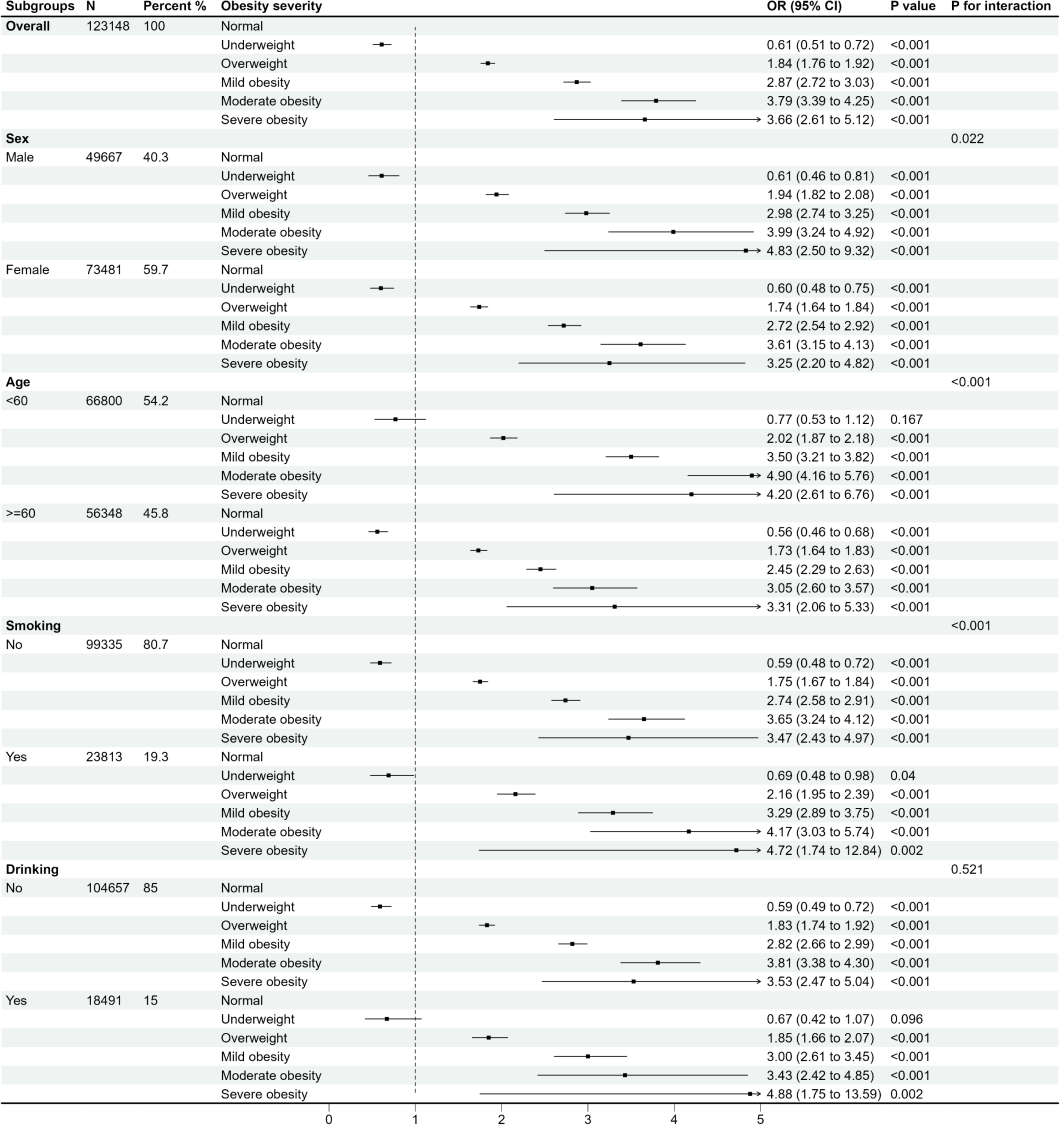
OR (95% CI) for the risk of multimorbidity according to different obesity severity by subgroups.

**Figure 4:**
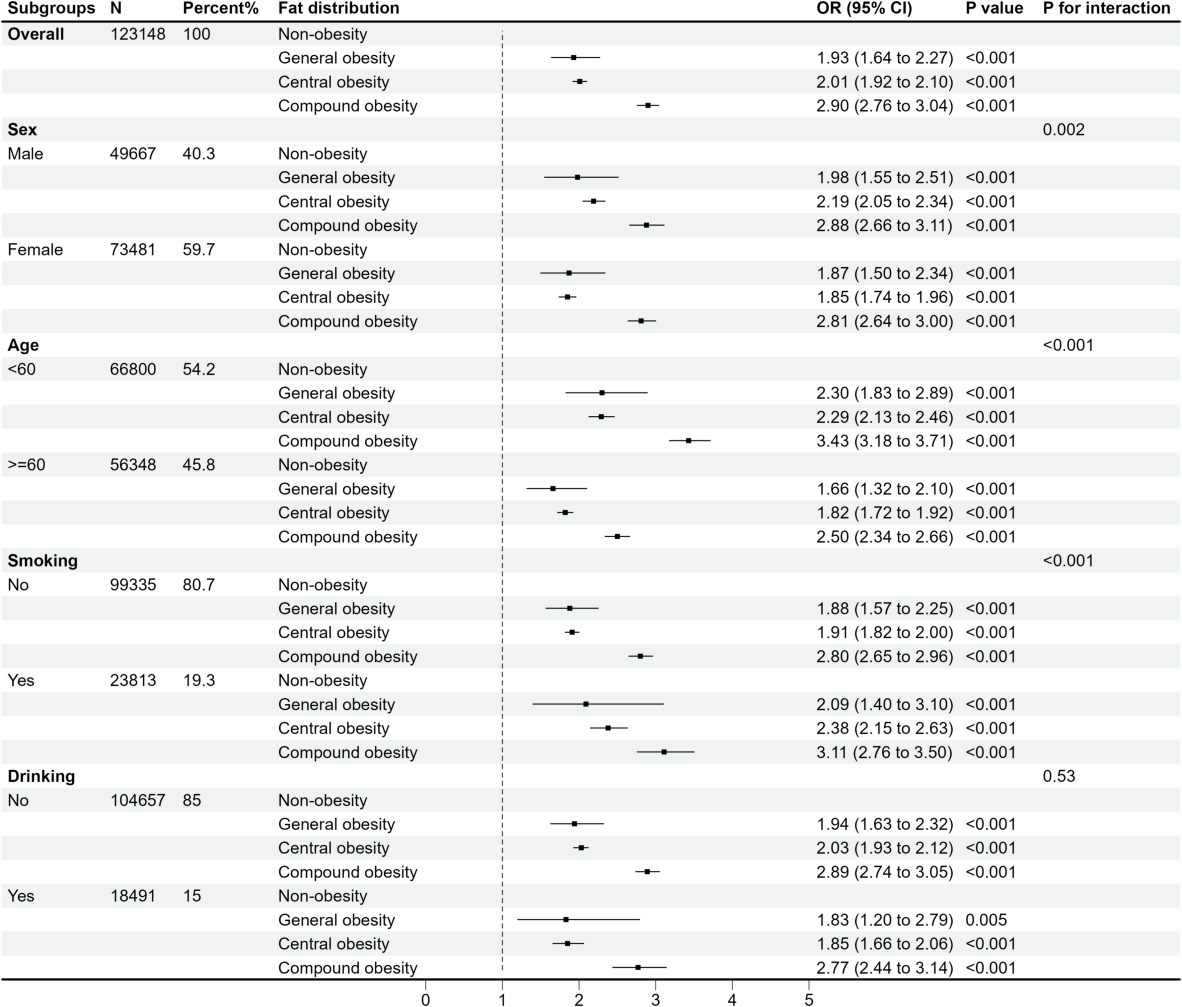
OR (95% CI) for the risk of multimorbidity according to different fat distribution by subgroups.

Subgroup and interaction analyses were performed by gender, age groups, smoking behavior, and alcohol drinking status. Stratified by different obesity severity (**Figure 3**) and fat distribution (**Figure 4**), the results revealed significant interaction effects among genders, age groups, and smoking statuses (*P* for interaction < 0.05). In contrast, drinking statuses showed no significant interactive effects (*P* for interaction > 0.05). Additionally, the males, people aged < 60 years, and smokers may worsen the effects of obesity on multimorbidity.

### 3.4 Association rules showing disease relationships in obesity severity and fat distribution

**Table 6** shows the ARM of disease relationships in different obesity severity by gender. Rules extracted through ARM were ranked by lift. In males, the most prominent associations emerge in severe obesity, where the combination of diabetes with either stroke or hypertension shows the strongest association to myocardial infarction (lift=16.0). The stroke-myocardial infarction association also appears notably strong (lift=10.7). For females, the most significant associations consistently involved diabetes, dyslipidemia, and hypertension, with lift values progressively increasing from mild obesity(lift=1.69) to severe obesity (lift=2.80). A clear gender difference appears in underweight individuals, where males show a relatively strong stroke-hypertension relationship (lift=1.29), while females display weaker associations in this category.

**Table 6:** Association rules showing disease relationships in different obesity severity by gender (sort by lift).

Rank	Male			Female		
	Antecedent	Consequent	Lift	Antecedent	Consequent	Lift
Normal						
1	Stroke	Hypertension	0.85	Diabetes	Dyslipidemia	1.07
2	Hypertension	Stroke	0.85	Dyslipidemia	Diabetes	1.07
3	Hypertension	Dyslipidemia	0.79	Stroke	Hypertension	0.91
Underweight						
1	Stroke	Hypertension	1.29	Stroke	Hypertension	0.71
2	Hypertension	Stroke	1.29	Hypertension	Stroke	0.71
3	COPD	Hypertension	0.46	Diabetes	Hypertension	0.56
Overweight						
1	Dyslipidemia	Diabetes	1.36	Diabetes Hypertension	Dyslipidemia	1.60
2	Diabetes	Dyslipidemia	1.36	Dyslipidemia Hypertension	Diabetes	1.56
3	Stroke	Hypertension	0.90	Dyslipidemia	Diabetes	1.23
Mild obesity						
1	Diabetes Hypertension	Dyslipidemia	1.92	Diabetes Hypertension	Dyslipidemia	1.69
2	Dyslipidemia Hypertension	Diabetes	1.82	Dyslipidemia Hypertension	Diabetes	1.64
3	Diabetes	Dyslipidemia	1.57	Diabetes	Dyslipidemia	1.39
Moderate obesity						
1	Diabetes Hypertension	Dyslipidemia	1.96	Diabetes Hypertension	Dyslipidemia	2.07
2	Dyslipidemia Hypertension	Diabetes	1.80	Dyslipidemia Hypertension	Diabetes	2.03
3	Dyslipidemia	Diabetes	1.60	Dyslipidemia	Stroke	1.95
Severe obesity						
1	Diabetes Stroke	Myocardial Infarction	16.0	Dyslipidemia Hypertension	Diabetes	2.80
2	Diabetes Hypertension Stroke	Myocardial Infarction	16.0	Dyslipidemia	Diabetes	2.62
3	Myocardial Infarction	Stroke	10.7	Diabetes	Dyslipidemia	2.62

The minimum number of combinations is limited to 2. They are sorted by lift and the top three disease combinations with the highest lift are displayed.

**Table 7** shows the ARM of disease relationships in different fat distribution by gender. In males, the strongest associations were observed in compound obesity, where the diabetes-hypertension combination shows a particularly strong link to dyslipidemia (lift=1.98), followed by central obesity (lift=1.81). The bidirectional relationship between diabetes and dyslipidemia remains consistently prominent across all obesity types in males, with progressively stronger associations from general to compound obesity. Similar to males, females exhibited consistent relationships between diabetes-hypertension and dyslipidemia across different fat distribution. Notably, in females, the most striking pattern appears in general obesity, where the diabetes-hypertension combination showed a strong association with dyslipidemia (lift=2.53).

**Table 7:** Association rules showing disease relationships in different fat distribution by gender (sort by lift).

Rank	Male			Female		
	Antecedent	Consequent	Lift	Antecedent	Consequent	Lift
Non-obesity						
1	Stroke	Hypertension	0.85	Dyslipidemia	Diabetes	1.13
2	Hypertension	Stroke	0.85	Diabetes	Dyslipidemia	1.13
3	Dyslipidemia	Hypertension	0.82	Stroke	Hypertension	0.88
General obesity						
1	Diabetes	Dyslipidemia	1.29	Diabetes Hypertension	Dyslipidemia	2.53
2	Dyslipidemia	Diabetes	1.29	Dyslipidemia Hypertension	Diabetes	2.42
3	Hypertension	Stroke	0.90	Dyslipidemia	Diabetes	1.71
Central obesity						
1	Diabetes Hypertension	Dyslipidemia	1.81	Diabetes Hypertension	Dyslipidemia	1.56
2	Dyslipidemia Hypertension	Diabetes	1.58	Dyslipidemia Hypertension	Diabetes	1.46
3	Dyslipidemia	Diabetes	1.31	Dyslipidemia	Diabetes	1.17
Compound obesity						
1	Diabetes Hypertension	Dyslipidemia	1.98	Diabetes Hypertension	Dyslipidemia	1.73
2	Dyslipidemia Hypertension	Diabetes	1.88	Dyslipidemia Hypertension	Diabetes	1.68
3	Dyslipidemia	Diabetes	1.60	Dyslipidemia	Diabetes	1.45

The minimum number of combinations is limited to 2. They are sorted by lift and the top three disease combinations with the highest lift are displayed.

## 4 Discussion

Utilizing a cross-sectional design, this investigation examined 123,148 adult participants (age range: 35–76 years) in 12 districts from Anhui Province, China. We examined multimorbidity patterns across different obesity severity and fat distribution, and identified characteristic disease combinations using ARM. Our results indicate that overweight and obesity independently contribute to multimorbidity risk, demonstrating a statistically significant dose-response relationship as obesity severity increases (*P* for trend < 0.001). Individuals with obesity are more vulnerable to multimorbidity involving diabetes, hypertension, and dyslipidemia. Central obesity, in particular, is significantly associated with higher risks of multimorbidity (OR=1.808, 95% CI 1.652-1.978, p<0.001) and warrants special attention in Chinese adults. Therefore, for individuals with varying obesity severity and fat distribution, effective intervention should include not only weight management but also waist circumference control and lifestyle changes, along with regular monitoring of blood pressure, lipids, and blood glucose to reduce the risk of multimorbidity.

Multimorbidity represents a pressing worldwide global health challenge due to population aging and rising chronic disease burdens. China is experiencing similar trends, with nearly one-quarter of adults affected by multimorbidity, and its prevalence increasing rapidly with age (14). Existing literature reports highly variable prevalence estimates (ranging from 6.4% to 76.5%) (15, 16), likely due to differences in study populations, geographic regions, and definitions or classifications of chronic conditions—factors that hinder direct comparisons across studies. In this study, we found that the community-based population in Anhui Province revealed a multimorbidity prevalence of 10.3% (n=12,644) across nine chronic diseases overall, with males (10.7%, n=5,324) showing slightly higher prevalence than females (9.96%, n=7,320). The prevalence increased to 20.5% (n=12,310) among adults aged ≥45 years and 29.1% (n=8,152) in the elderly population (≥60 years). These findings align with Fan et al.’s nationwide study (17), which reported a 15.8% prevalence among 500,000 Chinese adults, with age-stratified rates of 6.4% (<50 years) and 31.8% (≥60 years).

Numerous studies indicate that females are more likely to experience multimorbidity than males (18, 19), which is inconsistent with our research results. Specifically, our analysis revealed that males exhibited higher multimorbidity rates than females in the population under 60 years of age, whereas this relationship reversed among those aged ≥60 years. This discrepancy may be attributed to several factors. Males may initially be more vulnerable to engaging in unhealthy behaviors (e.g., tobacco use, alcohol consumption), but with the increase in age, postmenopausal females experience a decline in the protective effects of estrogen and progesterone, potentially increasing their susceptibility to multimorbidity (20, 21). Additionally, we observed that multimorbidity was more prevalent in the southern Anhui region, but did not vary by rural or urban residence. As shown in **Supplementary Table 2**, this region has an older population, lower health insurance coverage, and fewer high-income individuals compared to central and northern Anhui, with statistically significant differences. These demographic and socioeconomic differences likely contribute to the higher prevalence of multimorbidity, which is also consistent with other research findings (22).

Obesity substantially elevates the likelihood of developing various multiple chronic diseases, and evidence from the United States, Canada, and developing economies further underscores its critical role in increasing the risk of multimorbidity (23–25). In our study, after adjusting for multiple confounders, overweight individuals exhibited a 1.5-fold increased risk of multimorbidity (males: OR=1.454, 95% CI 1.320–1.600; females: OR=1.422, 95% CI 1.310–1.544). Individuals with obesity showed a 2- to 4-fold elevated risk compared to normal-weight counterparts (all p<0.001), and the risk of multimorbidity escalated with the severity of obesity (P for trend <0.001), which is aligned with previous research conclusions (26–28). Shan et al.’s systematic review (26) demonstrated 38% and 138% elevated likelihoods of multimorbidity among populations with overweight and obesity, respectively, revealing a dose-response relationship where each 1 kg/m² BMI increase correlated with 6% greater risk. Similarly, a nationally representative survey conducted in South Korea found a 63% elevated likelihood of multimorbidity associated with obesity (OR: 1.63, 95% CI: 1.45–1.84) (29). Furthermore, a Finnish prospective cohort study (28) found that the probability of multimorbidity increased progressively across obesity grades I, II, and III, with approximately 4.16-, 7.39-, and 12.43-fold higher risks compared to the control group, consistent with our findings.

BMI is a widely adopted clinical index for obesity assessment due to its standardization, simplicity, and practicality (30). However, its accuracy in evaluating body fat distribution and adiposity remains debated (31). Compared to BMI-defined overweight and obesity, WC-based measures of central and general obesity offer a more precise evaluation of fat distribution in the Chinese population (32). This investigation represents the inaugural examination of how different fat distribution correlate with multimorbidity. The results indicated that central obesity correlated with greater multimorbidity rates relative to general obesity, and exhibited a stronger effect in males (OR = 2.168, 95% CI: 1.857-2.532) than in females (OR = 1.567, 95% CI: 1.401-1.752). Moreover, individuals with compound obesity had the highest risk, demonstrating 2.223-fold (males) and 1.998-fold (females) greater likelihood of multimorbidity relative to participants with both healthy BMI and WC. These findings are consistent with prior research. For instance, a study of rural Brazilian workers (33) reported that increased WC significantly raised the odds of multimorbidity (OR = 2.82, 95% CI: 1.98-4.02). The Pró-Saúde study (34) also confirmed that higher BMI and WC are independently correlated with an increased risk of multimorbidity. Additionally, an analysis of NHANES 2011–2018 data (35) revealed a significant association between central fat distribution and multimorbidity in populations with obesity.

Association rule analysis revealed that the disease cluster comprising diabetes, hypertension, and dyslipidemia is most common in individuals with obesity, with the lift increasing alongside the severity of obesity. This clustering phenomenon can be attributed to the high population prevalence of these three conditions, which frequently co-occur as multimorbidity and mutually influence each other (36). Obesity may influence these three conditions through several interconnected mechanisms, including persistent inflammatory responses, oxidative damage, insulin resistance, and renin-angiotensin pathway stimulation (37). A longitudinal investigation of Japanese seniors (age ≥ 65 years) revealed a strong association between rising BMI and elevated rates of diabetes, hypertension, and dyslipidemia, with BMI confirmed as an autonomous predictor for these conditions (38). Additionally, research from Kenya reported significantly elevated multimorbidity risks associated with diabetes, hypertension, and dyslipidemia among participants with overweight (OR = 2.68, 95% CI: 1.72-4.18) and obesity (OR = 4.89, 95% CI: 2.94-8.14), with central obesity showing roughly quadruple the likelihood versus those with healthy WC (39). Consequently, we need to pay attention to and comprehensively manage hypertension, diabetes, and dyslipidemia multimorbidity to mitigate cardiovascular risk factors and reduce mortality from all causes.

Notably, among male participants, the most prominent associations emerge in severe obesity (BMI ≥ 37.5 kg/m²), where the combination of diabetes with either stroke or hypertension shows the strongest association to myocardial infarction (lift=16.0). The stroke-myocardial infarction association also appears notably strong (lift=10.7). These are characteristic features of cardiovascular metabolic comorbidity (CMM) (40). A cohort study involving 120,813 patients in Europe and the United States (41) reported individuals with BMI ≥ 35.0 kg/m² exhibited a tenfold greater CMM risk compared to normal-weight counterparts. Furthermore, according to the American Heart Association’s scientific reports (42), men aged 45–64 years exhibit a 2–3-fold higher risk of first myocardial infarction than age-matched women, while women over 75 experience comparable or even higher myocardial infarction and stroke risks than men. These established gender-specific differences were validated in our study population (aged 35–76), with association rules demonstrating a stronger male predisposition to CMM with severe obesity. Given that males have higher risks of multimorbidity than females across different obesity severity and fat distribution, physicians should prioritize enhanced health management for males with severe obesity in clinical practice, focusing on cardiovascular and metabolic diseases. Among female participants, the combination of diabetes-hypertension with dyslipidemia is most prominent in general obesity, and this may be attributable to distinct body fat distribution profiles between genders. Before menopause, women typically exhibit greater subcutaneous adipose tissue deposition, particularly in the buttocks and legs, whereas men predominantly accumulate visceral adipose tissue (43). Jiang et al. also found that (36), at the same BMI or WC levels, older females have a higher risk of hypertension, diabetes, and dyslipidemia than males. Therefore, for females with general obesity, even if their WC is within normal limits, clinical screening for blood pressure, blood glucose, and lipid levels should still be actively conducted.

This study incorporates notable strengths and certain limitations. For example, our research benefits from large sample size and represents the first investigation of multimorbidity patterns across different levels of obesity and fat distribution. However, our research has certain constraints. First, the study’s focus on residents of Anhui Province may restrict the applicability of results to diverse geographical areas and ethnic groups. Second, the reliance on self-reported data for chronic disease prevalence and lifestyle factors (including physical activity, adequate sleep and regular checkup), without objective clinical measurements or standardized assessment tools, may introduce recall bias and affect data accuracy. Third, our study did not collect detailed data on the quantity or types of food consumed by participants and did not investigate the presence of eating disorders, which could influence dietary-related interpretations. Furthermore, the dichotomization of education level, tobacco use, and alcohol consumption into binary categories may oversimplify these variables and reduce the granularity of the analysis. Finally, although BMI and WC were employed as standard obesity measures in this study, these indices have well-documented limitations in evaluating visceral adiposity and discriminating metabolically healthy obesity (44). Future studies should incorporate complementary assessments such as body fat percentage, waist-to-height ratio, and body roundness index to provide more comprehensive adiposity evaluation (45, 46).

This study highlights the importance of active weight management for individuals with overweight and obesity, as reducing obesity levels is associated with a lower burden of multimorbidity, particularly for conditions like diabetes, hypertension, and dyslipidemia. Notably, males exhibit significantly higher multimorbidity risks than females across all obesity categories, and males with severe obesity significantly increases cardiometabolic risk. For such patients, active weight management interventions must be implemented. Additionally, clinicians should pay particular attention to female patients with general obesity, as they have a higher risk of multimorbidity even if they appear outwardly healthy, underscoring the need for comprehensive clinical measures to assess obesity and guide personalized weight management strategies.

## Data Availability

The data analyzed in this study is subject to the following licenses/restrictions: For additional details, please contact the senior author. Requests to access these datasets should be directed to Keyi Gu, 1215491236@qq.com.

## References

[1] NCD Risk Factor Collaboration (NCD-RisC). Worldwide trends in underweight and obesity from 1990 to 2022: a pooled analysis of 3663 population-representative studies with 222 million children, adolescents, and adults. Lancet Lond Engl. (2024) 403:1027–50. doi: 10.1016/S0140-6736(23)02750-2, PMID: 38432237 PMC7615769

[2] Wang Y Zhao L Gao L Pan A Xue H. Health policy and public health implications of obesity in China. Lancet Diabetes Endocrinol. (2021) 9:446–61. doi: 10.1016/S2213-8587(21)00118-2, PMID: 34097869

[3] Gallagher EJ LeRoith D. Obesity and diabetes: the increased risk of cancer and cancer-related mortality. Physiol Rev. (2015) 95:727–48. doi: 10.1152/physrev.00030.2014, PMID: 26084689 PMC4491542

[4] Piché M-E Tchernof A Després J-P. Obesity phenotypes, diabetes, and cardiovascular diseases. Circ Res. (2020) 126:1477–500. doi: 10.1161/CIRCRESAHA.120.316101, PMID: 32437302

[5] Zheng C Liu Y Xu C Zeng S Wang Q Guo Y. Association between obesity and the prevalence of dyslipidemia in middle-aged and older people: an observational study. Sci Rep. (2024) 14:11974. doi: 10.1038/s41598-024-62892-5, PMID: 38796639 PMC11127928

[6] Jovic D Marinkovic J Vukovic D. Association between body mass index and prevalence of multimorbidity: a cross-sectional study. Public Health. (2016) 139:103–11. doi: 10.1016/j.puhe.2016.05.014, PMID: 27340043

[7] Skou ST Mair FS Fortin M Guthrie B Nunes BP Miranda JJ. Multimorbidity. Nat Rev Dis Primer. (2022) 8:48. doi: 10.1038/s41572-022-00376-4, PMID: 35835758 PMC7613517

[8] Zhang X Ha S Lau HC-H Yu J. Excess body weight: novel insights into its roles in obesity comorbidities. Semin Cancer Biol. (2023) 92:16–27. doi: 10.1016/j.semcancer.2023.03.008, PMID: 36965839

[9] Zhang J Xu L Li J Sun L Qin W. Association between obesity-related anthropometric indices and multimorbidity among older adults in Shandong, China: a cross-sectional study. BMJ Open. (2020) 10:e036664. doi: 10.1136/bmjopen-2019-036664, PMID: 32430453 PMC7239539

[10] Chen Z-T Wang X-M Zhong Y-S Zhong W-F Song W-Q Wu X-B. Association of changes in waist circumference, waist-to-height ratio and weight-adjusted-waist index with multimorbidity among older chinese adults: results from the chinese longitudinal healthy longevity survey (CLHLS). BMC Public Health. (2024) 24:318. doi: 10.1186/s12889-024-17846-x, PMID: 38287292 PMC10825986

[11] Zhang P Sun X Jin H Zhang F-L Guo Z-N Yang Y. Association between obesity type and common vascular and metabolic diseases: a cross-sectional study. Front Endocrinol. (2019) 10:900. doi: 10.3389/fendo.2019.00900, PMID: 31998234 PMC6962099

[12] Anari R Amani R Latifi SM Veissi M Shahbazian H. Association of obesity with hypertension and dyslipidemia in type 2 diabetes mellitus subjects. Diabetes Metab Syndr. (2017) 11:37–41. doi: 10.1016/j.dsx.2016.07.004, PMID: 27477531

[13] Li MY Luo YY Zhang P Chen W Zhang YQ Fang ZZ. interpretation of national clinical practice guideline on obesity management (2024 edition). Zhonghua Yi Xue Za Zhi. (2025) 105:1387–91. doi: 10.3760/cma.j.cn112137-20250221-00414, PMID: 40340216

[14] Hu Y Wang Z He H Pan L Tu J Shan G. Prevalence and patterns of multimorbidity in China during 2002-2022: a systematic review and meta-analysis. Ageing Res Rev. (2024) 93:102165. doi: 10.1016/j.arr.2023.102165, PMID: 38096988

[15] Nicholson K Liu W Fitzpatrick D Hardacre KA Roberts S Salerno J. Prevalence of multimorbidity and polypharmacy among adults and older adults: a systematic review. Lancet Healthy Longev. (2024) 5:e287–96. doi: 10.1016/S2666-7568(24)00007-2, PMID: 38452787

[16] Hu X Huang J Lv Y Li G Peng X. Status of prevalence study on multimorbidity of chronic disease in China: systematic review. Geriatr Gerontol Int. (2015) 15:1–10. doi: 10.1111/ggi.12340, PMID: 25163532

[17] Fan J Sun Z Yu C Guo Y Pei P Yang L. Multimorbidity patterns and association with mortality in 0.5 million chinese adults. Chin Med J (Engl). (2022) 135:648–57. doi: 10.1097/CM9.0000000000001985, PMID: 35191418 PMC9276333

[18] Yao S-S Cao G-Y Han L Chen Z-S Huang Z-T Gong P. Prevalence and patterns of multimorbidity in a nationally representative sample of older chinese: results from the China health and retirement longitudinal study. J Gerontol A Biol Sci Med Sci. (2020) 75:1974–80. doi: 10.1093/gerona/glz185, PMID: 31406983

[19] Abad-Díez JM Calderón-Larrañaga A Poncel-Falcó A Poblador-Plou B Calderón-Meza JM Sicras-Mainar A. Age and gender differences in the prevalence and patterns of multimorbidity in the older population. BMC Geriatr. (2014) 14:75. doi: 10.1186/1471-2318-14-75, PMID: 24934411 PMC4070347

[20] Liu JH. Multimorbidity in postmenopausal women: a new health challenge. Menopause N Y N. (2024) 31:943–4. doi: 10.1097/GME.0000000000002445, PMID: 39465993

[21] Jiao J Feng X Gong A Yao Y. Association between reproductive lifespan and multimorbidity among chinese postmenopausal women. Menopause N Y N. (2024) 31:945–51. doi: 10.1097/GME.0000000000002419, PMID: 39078652

[22] Zhao Y Zhao S Zhang L Haregu TN Wang H. Impacts of multimorbidity on medication treatment, primary healthcare and hospitalization among middle-aged and older adults in China: evidence from a nationwide longitudinal study. BMC Public Health. (2021) 21:1380. doi: 10.1186/s12889-021-11456-7, PMID: 34253222 PMC8274017

[23] Romano E Ma R Vancampfort D Firth J Felez-Nobrega M Haro JM. Multimorbidity and obesity in older adults from six low- and middle-income countries. Prev Med. (2021) 153:106816. doi: 10.1016/j.ypmed.2021.106816, PMID: 34599928

[24] Lebenbaum M Zaric GS Thind A Sarma S. Trends in obesity and multimorbidity in Canada. Prev Med. (2018) 116:173–9. doi: 10.1016/j.ypmed.2018.08.025, PMID: 30194961

[25] Pollack LM Wang M Leung MYM Colditz G Herrick C Chang S-H. Obesity-related multimorbidity and risk of cardiovascular disease in the middle-aged population in the United States. Prev Med. (2020) 139:106225. doi: 10.1016/j.ypmed.2020.106225, PMID: 32768511 PMC8008707

[26] Shan J Yin R Panuthai S. Body mass index and multimorbidity risk: a systematic review and dose-response meta-analysis. Arch Gerontol Geriatr. (2024) 123:105418. doi: 10.1016/j.archger.2024.105418, PMID: 38604087

[27] Agrawal S Agrawal PK. Association between body mass index and prevalence of multimorbidity in low-and middle-income countries: a cross-sectional study. Int J Med Public Health. (2016) 6:73–83. doi: 10.5530/ijmedph.2016.2.5, PMID: 28894693 PMC5591643

[28] Kivimäki M Strandberg T Pentti J Nyberg ST Frank P Jokela M. Body-mass index and risk of obesity-related complex multimorbidity: an observational multicohort study. Lancet Diabetes Endocrinol. (2022) 10:253–63. doi: 10.1016/S2213-8587(22)00033-X, PMID: 35248171 PMC8938400

[29] Association of sarcopenia and obesity with multimorbidity in korean adults: a nationwide cross-sectional study - PubMed . Available online at (Accessed June 17, 2025). 10.1016/j.jamda.2016.07.005 27567461

[30] Jensen MD Ryan DH Apovian CM Ard JD Comuzzie AG Donato KA. AHA/ACC/TOS guideline for the management of overweight and obesity in adults: a report of the american college of cardiology/american heart association task force on practice guidelines and the obesity society. Circulation. (2013) 129:S102–138. doi: 10.1161/01.cir.0000437739.71477.ee, PMID: 24222017 PMC5819889

[31] Low NY Chan CY Subramaniam S Chin K-Y Ima Nirwana S Muhammad N. Comparing the performance of body mass index, waist circumference and waist-to-height ratio in predicting Malaysians with excess adiposity. Ann Hum Biol. (2022) 49:299–304. doi: 10.1080/03014460.2022.2147585, PMID: 36373795

[32] Lear SA Lesser IA. A review of obesity and body fat distribution and its relationship to cardio-metabolic risk in men and women of chinese origin. Cardiovasc Hematol Disord Drug Targets. (2012) 12:113–8. doi: 10.2174/1871529x11202020113, PMID: 23030448

[33] Petarli GB Cattafesta M Sant’Anna MM Bezerra OM de PA Zandonade E Salaroli LB. Multimorbidity and complex multimorbidity in Brazilian rural workers. PloS One. (2019) 14:e0225416. doi: 10.1371/journal.pone.0225416, PMID: 31743369 PMC6863555

[34] Stumpf FM de M de Oliveira ASD Faerstein E Curioni CC. Cross-sectional associations between body mass index, waist circumference, and multimorbidity: pró-saúde study. PeerJ. (2023) 11:e14744. doi: 10.7717/peerj.14744, PMID: 36778147 PMC9910183

[35] Liu C-A Liu T Ruan G-T Ge Y-Z Song M-M Xie H-L. The relationship between fat distribution in central region and comorbidities in obese people: based on NHANES 2011-2018. Front Endocrinol. (2023) 14:1114963. doi: 10.3389/fendo.2023.1114963, PMID: 36843589 PMC9945539

[36] Jiang X Zhao Y Yang Q Wang W Lin T Qiu Y. Gender differences in associations between obesity and hypertension, diabetes, dyslipidemia: evidence from electronic health records of 3.5 million chinese senior population. BMC Public Health. (2025) 25:405. doi: 10.1186/s12889-025-21534-9, PMID: 39891113 PMC11786543

[37] Kotsis V Jordan J Micic D Finer N Leitner DR Toplak H. Obesity and cardiovascular risk: a call for action from the european society of hypertension working group of obesity, diabetes and the high-risk patient and european association for the study of obesity: part a: mechanisms of obesity induced hypertension, diabetes and dyslipidemia and practice guidelines for treatment. J Hypertens. (2018) 36:1427–40. doi: 10.1097/HJH.0000000000001730, PMID: 29634663

[38] Yamada T Kimura-Koyanagi M Sakaguchi K Ogawa W Tamori Y. Obesity and risk for its comorbidities diabetes, hypertension, and dyslipidemia in Japanese individuals aged 65 years. Sci Rep. (2023) 13:2346. doi: 10.1038/s41598-023-29276-7, PMID: 36759688 PMC9911391

[39] Temu TM Macharia P Mtui J Mwangi M Ngungi PW Wanjalla C. Obesity and risk for hypertension and diabetes among Kenyan adults: results from a national survey. Med (Baltimore). (2021) 100:e27484. doi: 10.1097/MD.0000000000027484, PMID: 34622879 PMC8500651

[40] Wan B Wang S Hu S Han W Qiu S Zhu L. The comprehensive effects of high-sensitivity C-reactive protein and triglyceride glucose index on cardiometabolic multimorbidity. Front Endocrinol. (2025) 16:1511319. doi: 10.3389/fendo.2025.1511319, PMID: 40235659 PMC11996647

[41] Kivimäki M Kuosma E Ferrie JE Luukkonen R Nyberg ST Alfredsson L. Overweight, obesity, and risk of cardiometabolic multimorbidity: pooled analysis of individual-level data for 120 813 adults from 16 cohort studies from the USA and Europe. Lancet Public Health. (2017) 2:e277–85. doi: 10.1016/S2468-2667(17)30074-9, PMID: 28626830 PMC5463032

[42] Arnett DK Blumenthal RS Albert MA Buroker AB Goldberger ZD Hahn EJ. ACC/AHA guideline on the primary prevention of cardiovascular disease: executive summary: A report of the american college of cardiology/american heart association task force on clinical practice guidelines. Circulation. (2019) 140:e563–95. doi: 10.1161/CIR.0000000000000677, PMID: 30879339 PMC8351755

[43] Koceva A Herman R Janez A Rakusa M Jensterle M. Sex- and gender-related differences in obesity: from pathophysiological mechanisms to clinical implications. Int J Mol Sci. (2024) 25:7342. doi: 10.3390/ijms25137342, PMID: 39000449 PMC11242171

[44] Sweatt K Garvey WT Martins C. Strengths and limitations of BMI in the diagnosis of obesity: what is the path forward? Curr Obes Rep. (2024) 13:584–95. doi: 10.1007/s13679-024-00580-1, PMID: 38958869 PMC11306271

[45] Flegal KM Shepherd JA Looker AC Graubard BI Borrud LG Ogden CL. Comparisons of percentage body fat, body mass index, waist circumference, and waist-stature ratio in adults. Am J Clin Nutr. (2009) 89:500–8. doi: 10.3945/ajcn.2008.26847, PMID: 19116329 PMC2647766

[46] Oliveira BR Magalhães EI da S Bragança MLBM Coelho CCN da S NP L Bettiol H. Performance of body fat percentage, fat mass index and body mass index for detecting cardiometabolic outcomes in Brazilian adults. Nutrients. (2023) 15:2974. doi: 10.3390/nu15132974, PMID: 37447300 PMC10346298

