# Supplementary material for: Patterns of multimorbidity across obesity severity and fat distribution in Anhui, China: a community-based study
Source: Front Endocrinol (Lausanne). 2025 Sep 10;16:1652678. doi: 10.3389/fendo.2025.1652678 (PMC12457183; doi:10.3389/fendo.2025.1652678)
Supplement: Supplementary file 1 [file DataSheet1.pdf]

Supplementary Material

Supplementary Table 1 Baseline characteristics of participants by gender

Parameters	Overall	Female	Male	*P* value
Number of participants (%)	123148	73481（59.7%）	49667（40.3%）	
Multimorbidity (%)	12644（10.3%）	7320（9.96%）	5324（10.7%）	
**Socio-demographic factors**				
Age (years)	57.71±10.01	57.07±9.87	58.66±10.15	<0.001
Age groups (%)				<0.001
(35-45]	15342 (12.5%)	9459 (12.9%)	5883 (11.8%)	
(45-60]	54060 (43.9%)	34398 (46.8%)	19662 (39.6%)	
≥60	53746 (43.6%)	29624 (40.3%)	24122 (48.6%)	
Region (%)				<0.001
Northern Anhui	32298 (26.2%)	20346 (27.7%)	11952 (24.1%)	
Central Anhui	38836 (31.5%)	22907 (31.2%)	15929 (32.1%)	
Southern Anhui	52014 (42.2%)	30228 (41.1%)	21786 (43.9%)	
Rural (%)	78132 (63.4%)	47608 (64.8%)	30524 (61.5%)	<0.001
Married (%)	111292 (90.4%)	65442 (89.1%)	45850 (92.3%)	<0.001
High school or above (%)	12805 (10.4%)	5721 (7.8%)	7084 (14.3%)	<0.001
Annual income≥50k (%)	24672 (20.0%)	13835 (18.8%)	10837 (21.8%)	<0.001
Have insurance (%)	122490 (99.5%)	73080 (99.5%)	49410 (99.5%)	0.530
**Lifestyle factors**				
Smoking (%)	23813 (19.3%)	1197 (1.6%)	22616 (45.5%)	<0.001
Drinking (%)	18491 (15.0%)	2539 (3.5%)	15952 (32.1%)	<0.001
Rational diet (%)	36478 (29.6%)	22758 (31.0%)	13720 (27.6%)	<0.001
Weight control (%)	55907 (45.4%)	33821 (46.0%)	22086 (44.5%)	<0.001
Physical exercise (%)	74995 (60.9%)	45895 (62.5%)	29100 (58.6%)	<0.001
Adequate sleep (%)	31665 (25.7%)	19700 (26.8%)	11965 (24.1%)	<0.001
Regular checkup (%)	26132 (21.2%)	15912 (21.7%)	10220 (20.6%)	<0.001
**Clinical indicators**				
BMI	24.57±3.37	24.64±3.48	24.47±3.21	<0.001
WC (cm)	84.13±9.40	82.98±9.32	85.83±9.25	<0.001
SBP (mmHg)	136.94±19.66	136.55±20.32	137.52±18.62	<0.001
DBP (mmHg)	80.49±10.71	79.10±10.46	82.54±10.74	<0.001
HR (bpm)	73.73±10.09	74.46±9.79	72.65±10.43	<0.001
TC (mmol/L)	4.59±1.01	4.75±1.03	4.37±0.95	<0.001
LDL-c (mmol/L)	2.43±0.86	2.52±0.88	2.31±0.83	<0.001
HDL-c (mmol/L)	1.48±0.40	1.53±0.39	1.41±0.40	<0.001

TG (mmol/L)	1.57±0.82	1.60±0.80	1.51±0.84	<0.001
FPG (mmol/L)	6.11±1.74	6.10±1.74	6.13±1.73	<0.001

Mean ± SD for continuous variables: the p-value was calculated by the weighted linear regression model. (%) for categorical variables: the p-value was calculated by the weighted chi-square test. BMI, body mass index; WC, waist circumference; SBP, systolic blood pressure; DBP, diastolic blood pressure; HR, heart rate; TC, total cholesterol; LDL-c, low-density lipoprotein cholesterol; HDL-c, high-density lipoprotein cholesterol; TG, triglycerides; FPG, fasting blood glucose.

Supplementary Table 2 Demographic characteristics by region in Anhui Province, China

**Parameters**	**Northern Anhui**	**Central Anhui**	**Southern Anhui**	***P* value**
Age (years)	56.15±9.76	57.86±10.07	58.57±10.01	<0.001
Rural (%)	82.3	59.7	54.5	<0.001
Female (%)	63.0	59.0	58.1	<0.001
Married (%)	91.1	90.3	90.0	<0.001
High school or above (%)	9.6	4.2	15.6	<0.001
Annual income≥50k (%)	15.0	23.1	20.9	<0.001
Have insurance (%)	99.7	99.7	99.1	<0.001
Multimorbidity (%)	10.8	7.8	11.7	<0.001

Mean ± SD for continuous variables: the p-value was calculated by the weighted linear regression model. (%) for categorical variables: the p-value was calculated by the weighted chi-square test.
