# Supplementary figures and images for: Patterns of multimorbidity across obesity severity and fat distribution in Anhui, China: a community-based study
Source: Front Endocrinol (Lausanne). 2025 Sep 10;16:1652678. doi: 10.3389/fendo.2025.1652678 (PMC12457183; doi:10.3389/fendo.2025.1652678)

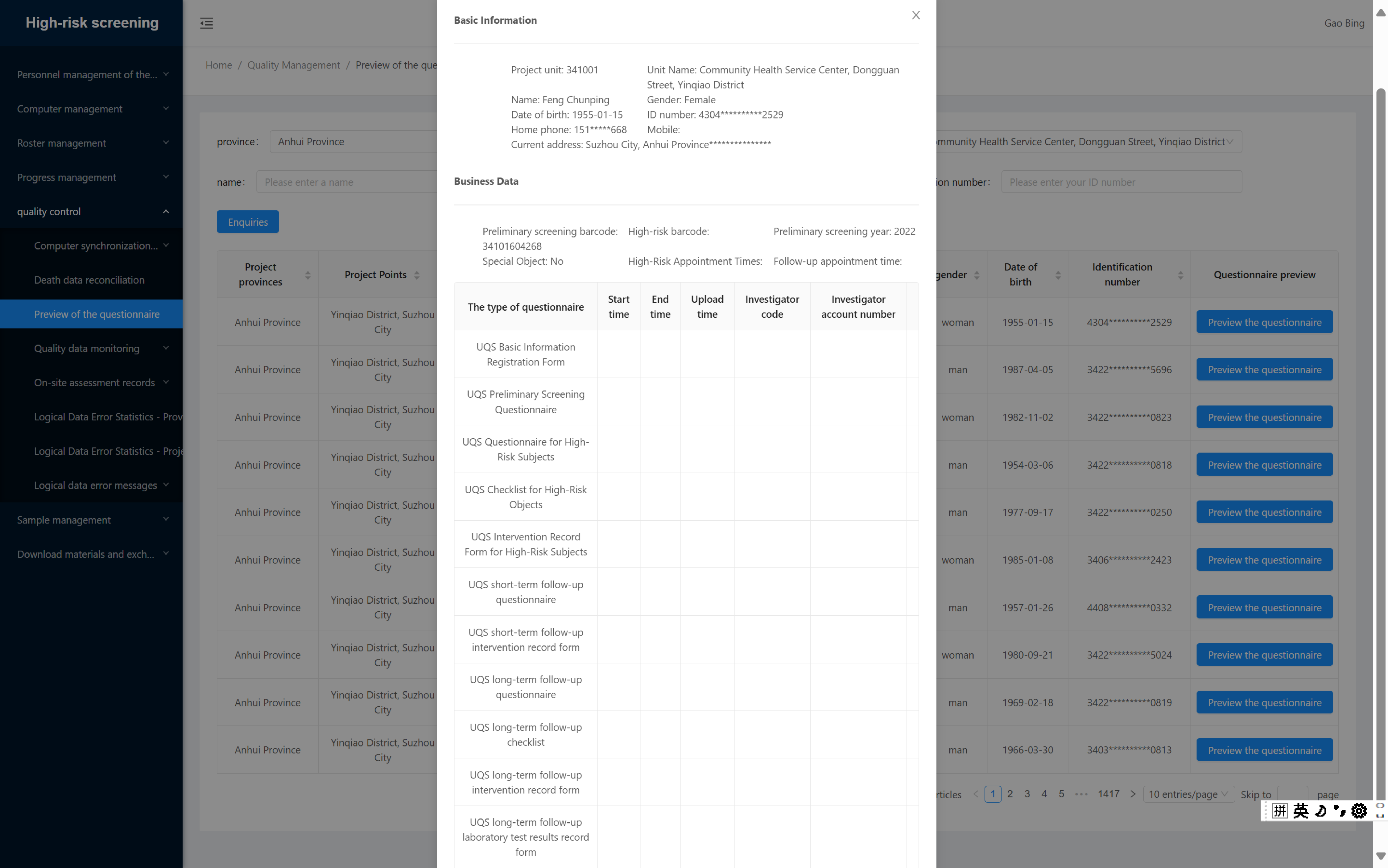

Supplement: Supplementary file 2 [file Image1.tif]
